# Measuring General Health Literacy in Chinese adults: validation of the HLS_19_-Q12 instrument

**DOI:** 10.1186/s12889-024-17977-1

**Published:** 2024-04-15

**Authors:** Rongmei Liu, Qiuping Zhao, Mingyang Yu, Hui Chen, Xiaomo Yang, Shuaibin Liu, Orkan Okan, Xinghan Chen, Yuhan Xing, Shuaijun Guo

**Affiliations:** 1 Hypertension Prevention and Treatment Centre of Henan Province, Fuwai Central China Cardiovascular Hospital, Zhengzhou, China; 2 Community Health Centre of Chaohe, Zhengzhou, China; 3 School of Medicine and Health, Technical University of Munich, Munich, Germany; 4 College of Basic Medicine and Forensic Medicine, Henan University of Science and Technology, Luoyang, China; 5 School of Public Health, Zhengzhou University, Zhengzhou, China; 6 Centre for Community Child Health, Murdoch Children’s Research Institute, Melbourne, Australia; 7 Department of Pediatrics, University of Melbourne, Melbourne, Australia

**Keywords:** Health literacy measurement, Validation, China, HLS_19_-Q12, Adults

## Abstract

**Background:**

Health literacy measurement lays a solid foundation to identify associations with health outcomes and monitor population health literacy levels over time. In mainland China, most existing health literacy instruments are either knowledge-based or practice-based, making health literacy results incomparable between China and other countries. This study aimed to examine the reliability and validity of the 12-item Health Literacy Population Survey (HLS_19_-Q12) in a general population of Chinese adults.

**Methods:**

A cross-sectional study was conducted to recruit primary carers of students from 11 schools in Zhengzhou, Henan Province, using convenience cluster sampling. Participants completed an online self-administered survey that collected information on key sociodemographics, health literacy (HLS_19_-Q12 and a comparison tool: Health Literacy Questionnaire (HLQ)), and health-related outcomes. Using the COnsensus-based Standards for the selection of health status Measurement Instruments (COSMIN) checklist as a guideline, we tested internal consistency, test-retest reliability, content validity, structural validity, concurrent predictive validity, and convergent validity of the HLS_19_-Q12.

**Results:**

Overall, 14,184 participants completed the full survey. The HLS_19_-Q12 showed excellent internal consistency (Cronbach’s α = 0.93), moderate test-retest reliability (intra-class correlation coefficient = 0.54), satisfactory content validity (based on the 12-matrix health literacy model), and strong structural validity (comparative fit index = 0.94, Tucker and Lewis’s index of fit = 0.93, root mean square error of approximation = 0.095). Concurrent predictive validity results showed health literacy was associated with both health determinants and health-related outcomes. The HLS_19_-Q12 had weak to strong correlations (coefficients = 0.24 to 0.42) with the nine scales of the HLQ. Respondents had an average score of 81.6 (± 23.0) when using the HLS_19_-Q12, with 35.0% and 7.5% having problematic and inadequate levels of health literacy, respectively.

**Conclusions:**

The HLS_19_-Q12 is a reliable and valid instrument to measure health literacy in our sample. Further validation is needed with a more nationally representative sample of Chinese adults. The HLS_19_-Q12 could be used as a comprehensive, skills-based, and easy-to-administer health literacy assessment tool integrated into population surveys and intervention evaluations.

**Supplementary Information:**

The online version contains supplementary material available at 10.1186/s12889-024-17977-1.

## Background

Health literacy is a key concept in public health [1]. Since its inception, the term “health literacy” has been widely used in the fields of health care, disease prevention, and health promotion [2]. While there is more than one definition, health literacy generally refers to an individual’s ability to find, understand, evaluate, and use health information to maintain and promote health over the life course [3]. Similar to income, education, and race, health literacy is regarded as a key determinant of health [4]. Findings from systematic reviews show that low health literacy is associated with a range of negative health outcomes, including poor health status, health-compromising behaviours, and high healthcare costs [5, 6]. To improve population health and reduce health inequities, many national governments and international organizations have integrated health literacy into their health plans, agendas, and initiatives [7–9].

Health literacy measurement lays a solid foundation to identify associations with outcomes of interest and monitor health literacy levels over time in a given population [10]. However, health literacy is multi-dimensional, making it difficult to measure and compare across different populations and contexts. Currently, there are more than 270 health literacy instruments developed and tested globally, with a focus on health-related skills assessment for both individuals and populations [11]. In Europe, the most commonly-used health literacy instruments are the European Health Literacy Survey Questionnaire (HLS-EU-Q47) [12], and its derived versions (e.g., HLS-EU-Q16, the HLS-EU-Q12, and HLS-EU-Q6) [13, 14]. In North America, health literacy measurement tools include the Short Assessment of Health Literacy (SAHL), Rapid Estimate of Adult Literacy in Medicine (REALM), and the National Assessment of Adult Literacy [15, 16]. In Australia, the Health Literacy Questionnaire (HLQ) is widely used to assess the needs and challenges of a wide range of populations [17]. All these measurement tools are useful in clinical interviews, population surveys, intervention evaluations, and policy decisions.

Health literacy has gained increasing attention in mainland China since 2008, when the Chinese National Health Commission issued a public bulletin that introduced the “Chinese Resident Health Literacy-Basic Knowledge and Skills” [8]. Based on this bulletin, the Chinese Health Literacy Scale (CHLS) was developed that contained three domains in terms of basic knowledge and attitudes, healthy behaviours and lifestyles, and health-related skills [18]. Since then, the CHLS has been widely used to examine and monitor population health literacy at national, provincial and local levels [18]. However, the CHLS mainly focuses on measuring health knowledge and practices, making health literacy results difficult to compare between mainland China and other western countries in the global context, which focus on measuring health-related skills [19–21].

Recently, the World Health Organisation (WHO) Action Network for Measuring Population and Organizational Health Literacy (M-POHL) has adapted the original HLS-EU-Q12 to a new health literacy instrument [22], a 12-item Health Literacy Population Survey Questionnaire (HLS_19_-Q12). Compared to the original HLS-EU-Q12, the HLS_19_-Q12 had changes in the wording of response categories (i.e., “very easy, fairly easy, fairly difficult, and very difficult” were changed to “very easy, easy, difficult, and very difficult”). In addition, out of the 12 items of the HLS_19_-Q12, 11 items were modified, including rewording of items as well as adding or removing examples from individual items, in order to better represent the underlying 12-matrix model that has three domains (health care, disease prevention, health promotion) and four specific health literacy areas (access, understand, evaluate, and apply health information). The HLS_19_-Q12 not only measures general health literacy comprehensively, but also shows satisfactory psychometric properties and high feasibility (e.g., self-administered, time-efficient) in population surveys. The HLS_19_-Q12 has been tested in more than 20 countries and found as a suitable instrument to measure health literacy across different cultural and linguistic contexts [22, 23].

While there have been several skills-based health literacy instruments tested in Chinese adults over the last decade [24] (e.g., HLQ [25], the Short Test of Functional Health Literacy in Adults [26], health literacy assessment regarding infectious respiratory diseases [27], health literacy competencies for health professionals [28]), these instruments either measure health literacy with single domains/areas, or target a specific population, or have administrative and respondent burdens (e.g., long time to complete). There remains a need to develop a time-efficient, skills-based, and comprehensive health literacy instrument that can be used in the general Chinese population. To address this gap, the present study aims to psychometrically evaluate the HLS_19_-Q12 in a general population of Chinese adults.

## Methods

### Participants and settings

A cross-sectional study was designed to recruit primary carers of students from 11 schools in Zhengzhou, Henan Province, China, using convenience cluster sampling. Zhengzhou is the capital of Henan, which has a population of 1.28 million in 2022. In brief, two districts (urban/rural) were selected according to their socioeconomic levels, one representing high and the other representing low. Based on previous successful collaborations with schools, we selected five or six schools in each district (urban: three primary schools and two middle schools; rural: four primary schools and two middle schools) according to the appropriateness of survey timing (class time or class break time). At each school, all primary carers of students in the whole class (ranging from 30 to 60 students) from each of the year levels (Year 1 to 6 or Year 7 to 8) were invited to complete an online self-administered questionnaire via Wenjuanxing. Participants were informed about the research aim and methods before signing the informed online consent form. Written informed consent was obtained from all subjects prior to filling out the questionnaire. Based on the measurement theory, the minimum sample size required was 400 [29]. Data collection was undertaken between September 2022 and June 2023. We used the Strengthening the Reporting of Observational Studies in Epidemiology (STROBE) checklist [30] and the COnsensus-based Standards for the selection of health status Measurement INstruments (COSMIN) checklist [31] as guidelines to ensure the reporting quality of the present study.

### Questionnaire

A Chinese version questionnaire was designed based on the research purpose to collect information on health literacy, key sociodemographics, and health outcomes. In total, there were three parts in this questionnaire (Part 1: You and Your Family; Part 2: Health Literacy; Part 3: Your Personal Health), with each part having 13 to 44 questions. A schoolteacher in each class sent the survey link to the primary carer of each student via the school information system. The average time to complete the survey was 15 min.

### Sociodemographics

We collected socio-demographic information on participants’ sex (male/female), age (35 years or below/36–40 years/41 years or above), ethnicity (Han/ethnic minorities), single-parent families (yes/no), highest educational level (Year 6 or below/Year 7–9/Year 10–12/Diploma/Bachelor’s degree or above), and household annual income (20,000 CNY or below/20,000 to 39,000 CNY/40,000 to 59,000 CNY/60,000 to 79,000 CNY/80,000 to 149,000 CNY/150,000 CNY or above).

### Health literacy assessment

Two self-report health literacy instruments were used in the present study: the HLS_19_ -Q12 and the full version of the 44-item HLQ. The HLQ was selected as a comparison tool to test convergent validity of the HLS_19_ -Q12 because it measured health literacy comprehensively and was available in Chinese. We hypothesised that there would be positive associations between their health literacy scores. We first translated the HLS_19_ -Q12 from English to Chinese according to Beaton’s guidelines [32], which includes two forward translations, a synthesis of forward translation, one backward translation, a translation committee review, and a pilot test with ten parents to ensure the readability and clarity of each item. Compared to the original HLS_19_ -Q12, we made several minor changes to ensure its appropriateness in Chinese culture (see Appendix 1 for details).

The HLS_19_ -Q12 is a 12-item health literacy instrument developed by the WHO M-POHL working group that measures comprehensive, general health literacy in general adult populations [22]. The HLS_19_ -Q12 is 12-matrix (three health domains×four health management tasks), which comprises of three health domains (health care, disease prevention, and health promotion) and four aspects of health information management (access, understand, evaluate, and apply). Respondents answered each item on a four-point Likert scale (1 = very difficult, 4 = very easy) concerning the experienced difficulty of each task. The total score of the HLS_19_ -Q12 was calculated as the percentage (ranging from 0 to 100) of items with valid responses that were answered with “very easy” or “easy” provided that at least 80% of the items contain valid responses [22]. If less than 80% of the items contain valid responses, the score was coded as “missing.” Higher scores of the HLS_19_ -Q12 indicate higher levels of health literacy. A categorical variable of the HLS_19_ -Q12 was also calculated to show the population distribution of health literacy. Four categories were created based on the recommended cut-off points by the WHO M-POHL working group [22]: (1) Excellent: “very easy” ≥ 50 and “very difficult” + “difficult” < 8.334, the number of answers with “very easy” should be above ½ and the answers for “very difficult” + “difficult” should be no more than 1/12; (2) Sufficient: “very easy” + “easy” > 83.33, at least 10 out of the 12 items should be answered with “very easy” or “easy” and not more than 2 out of 12 with “very difficult” or “difficult”; (3) Problematic: all respondents who were not in groups of “excellent”, “sufficient”, or “inadequate”; and (4) Inadequate: “very easy” < 8.334, “very difficult” and “difficult” ≥ 50, the number of answers with “very difficult” + “difficult” should be above ½ and for “very easy” should be no more than 1/12.

The HLQ is a comprehensive 44-item health literacy instrument that assess the needs and challenges of populations [17]. The HLQ consists of nine scales (Scale 1: Feeling understood and supported by healthcare providers, Scale 2: Having sufficient information to manage my health, Scale 3: Actively managing my health, Scale 4: Social support for health, Scale 5: Appraisal of health information, Scale 6: Ability to actively engage with healthcare providers, Scale 7: Navigating the healthcare system, Scale 8: Ability to find good health information, Scale 9: Understand health information well enough to know what to do), with each scale having four to six items that are scored on a Likert scale [17]. Respondents answered each item in Scales 1 to 5 with four responses (1 = strongly disagree, 4 = strongly agree) and each item in Scale 6 to 9 with five responses (1 = cannot do or always difficult, 5 = always easy) [17]. If less than 50% of the items within each scale contain valid responses, the scale score was coded as “missing.” Average scores of each scale were calculated by summing item scores and dividing by the number of items within that scale, with higher scores indicating higher health literacy. The HLQ is available in Chinese version and shows strong validity and high reliability [25].

### Health outcomes

We tested concurrent predictive validity of the HLS_19_ -Q12 by quantifying the association between the total scores of the HLS_19_ -Q12 and three health outcomes (health status, health-compromising behaviors, and health service use).

Health status was measured by a widely used general self-report health question (‘In general, would you say your health is?’ 1 = excellent, 5 = poor), which has demonstrated strong predictive validity with objective indicators of health and mortality [33]. Health status scores ranged from 1 to 5, with higher scores indicating poorer health status.

Health-compromising behaviours were measured by three items [34], which included cigarette smoking (“Are you smoking?”; 1 = currently; 2 = ever; 3 = never), alcohol drinking (“Have you had any alcohol in the past 30 days? (more than half a bottle or a can of beer, a small cup of spirit, a glass of wine or yellow wine)”; 1 = yes; 2 = no) and physical exercise frequency (“How many times have you exercised for 30 minutes or more in the past 30 days, such as running, walking, cycling, etc?”; 1 = almost no; 2 = several times a month; 3 = several times a week; 4 = almost every day). A total score of health-compromising behaviours is obtained by reversing and summing scores across all three items, with higher scores (ranging from 3 to 9) indicating more health-compromising behaviours.

Health service use was assessed by four items [35], which asked respondents to report the frequency of emergency service use (‘how many times have you used the emergency service in the last 12 months?’; 1 = 0 times, 4 = 6 times or more), general practitioner service use (‘how many times have you been to see a general practitioner in the last 12 months?’; 1 = 0 times, 4 = 6 times or more), hospitalisation (‘how many times have you stayed in a hospital in the last 12 months?’; 1 = 0 times, 4 = 6 times or more) and patient-provider communication (‘how many times have you raised a question during your doctor’s appointment in the last 12 months?’; 1 = 0 times, 4 = 6 times or more). A total score of health service use is obtained by recoding the score on each item (1 = 0, 2 = 1, 3 = 2, 4 = 3) and then summing scores across all four items. The total score of health service use ranged from 0 to 12, with higher scores indicating more use of health services.

### Statistical analysis

Our analytic sample consisted of all respondents who had valid data on the HLS_19_ -Q12. In the present study, 16,187 respondents participated in the online questionnaire, with 2003 respondents having incomplete data on health literacy. Therefore, our analytic sample consisted of 14,184 respondents, with a response rate of 87.6% (14,184/16,187). All analyses were undertaken using Stata 18.0 [36]. Descriptive statistics were conducted to show the distribution of participants’ characteristics, health-related outcomes, and the HLS_19_ -Q12 scores (each item and total). Internal consistency was examined by calculating Cronbach’s α, with more than 0.7 indicating satisfactory internal consistency [37]. Test-retest reliability was calculated by the intra-class correlation coefficient (ICC), with 0.75–0.90, 0.50–0.75 and less than 0.50 indicating high, moderate, and low test-retest reliability [38].

Content validity was judged by the translation committee about the relevance and comprehensiveness of each item for the construct of interest [31]. Structural validity was tested by confirmative factor analysis (CFA). A CFA model was considered adequate if the comparative fit index (CFI) value exceeded 0.9, the Tucker and Lewis’s index of fit (TLI) value exceeded 0.9, the root mean square error of approximation (RMSEA) value was below 0.1 [3]. Following the similar methodology used by Pelikan et al. [22] and previous empirical findings [12, 39], we tested concurrent predictive validity in terms of associations with social determinants of health and health consequences. We hypothesised that there would be a social gradient of health literacy when evaluating the linear regression model with the total score of the HLS_19_ -Q12 as the outcome variable and sex, age, ethnicity, highest educational level, and household income as predictor variables. Regarding health consequences, we hypothesised that health literacy would be associated with health status, health-compromising behaviours, and health service use, after adjusting for sex, age, ethnicity, highest educational level, and household income. Finally, convergent validity was examined by Pearson correlation between the total scores of the HLS_19_ -Q12 and average scores of each scale of the HLQ. The strength of relationship was considered as weak, moderate, strong if the correlation coefficient was less than 0.3, 0.3–0.4 and more than 0.4 respectively [40].

### Missing data

The proportion of respondents with complete data across all study variables was 68.8% (9756/14,184) (see Appendix 2). To examine the potential impact of missing data, we used multiple imputation by chained equations to reduce the potential bias due to incomplete records [41]. The imputation model included all study variables and one auxiliary variable (occupation). Based on the percentage of missing data [41], we produced 30 imputed datasets and used Rubin’s rules to obtain the final imputed estimates of the parameters of interest. Results using multiply imputed data are similar to those using the complete case data for all association analyses (see Appendix 3), suggesting that findings are likely to be valid even in the presence of missing data, therefore we report results using the complete case dataset in the main text.

## Results

### Participants’ characteristics

Of 14,184 respondents, the mean age of participants was 38.1 (± 5.7) years (age range = 22 to 77 years). Table 1 shows the distribution of sex, age group, ethnicity, single parenthood, highest educational level, household income, and geographic location. Compared to those who did not complete the HLS_19_-Q12, we found that those who were female, older, less educated, had lower income and lived in rural areas were likely to be missed out (see Appendix 4). Among those who had missing data on the HLS_19_-Q12 (*n* = 2003), the percentage of participants with missing data on a specific item ranged from 18.9% (item 4 “to act on advice from your doctor or pharmacist?”) to 71.6% (item 3 “to judge the advantages and disadvantages of different treatment options”) (see Appendix 4).

**Table 1:** Summary of participants’ characteristics and health outcomes (*N* = 14,184)

Variable	n (%) / mean(SD)
Sex	
Female	9863 (69.5)
Male	4321 (30.5)
Age	38.1 (5.7)
Age group	
35 years or below	4647 (36.2)
36 to 40 years	4661 (36.3)
41 years or above	3536 (27.5)
Ethnicity	
Han	13,816 (97.4)
Ethnic minorities	368 (2.6)
Single parenthood	
No	13,487 (95.1)
Yes	697 (4.9)
Highest educational level	
Year 6 or below	556 (3.9)
Year 7–9	5138 (36.2)
Year 10–12	3581 (25.2)
Diploma	2893 (20.4)
Bachelor’s degree or above	2016 (14.2)
Household income	
20,000 CNY or below	2833 (26.6)
20,000 to 39,000 CNY	2088 (19.6)
40,000 to 59,000 CNY	1867 (17.5)
60,000 to 79,000 CNY	1356 (12.7)
80,000 to 149,000 CNY	1590 (14.9)
150,000 CNY or above	908 (8.5)
Geographic location	
Urban	4013 (28.3)
Rural	10,171 (71.7)
Health status (continuous, 1–5)	2.01 (0.83)
Health compromising behaviours (continuous, 3–9)	5.07 (1.38)
Health service use (continuous, 0–12)	1.68 (1.71)

SD, standard deviation

### Item definition and descriptive results of the HLS_19_-Q12 instrument

Table 2 shows the descriptive result for each item and the total scores of the HLS_19_-Q12. The percentage of participants answering “very difficult” or “difficult” ranged from 8.4 to 40.3% across the 12 items, with item 10 (“to understand advice concerning your health from family or friends”) being the easiest item and item 3 (“to judge the advantages and disadvantages of different treatment options”) being the most difficult. The overall percentage of participants who answered “very difficult” or “difficult” responses for at least one item was 61.0%. The average of total health literacy scores was 81.6 (± 23.0). According to the pre-defined cut-offs for the HLS_19_ -Q12 in the Methods section, we found 20.7% and 36.8% of respondents had excellent and sufficient levels of health literacy respectively. On the other hand, 35.0% and 7.5% of respondents had problematic and inadequate levels of health literacy, respectively.

**Table 2:** Descriptive results of the HLS_19_-Q12 instrument (*N* = 14,184)

Item number	Question: “On a scale from very easy to very difficult, how easy would you say it is…”	Mean (± SD) / n (%)	Percentages of “very difficult” or “difficult” responses (%)
1	to find out where to get professional help when you are ill? (e.g., doctor, pharmacist, psychologist)	1.7 (± 0.7)	9.0
2	to understand information about what to do in a medical emergency (e.g., fainting, coma, trauma)?	2.1 (± 0.7)	29.5
3	to judge the advantages and disadvantages of different treatment options (e.g., surgery, Western medicine, Chinese medicine)?	2.3 (± 0.8)	40.3
4	to act on advice from your doctor or pharmacist? (e.g., eat less fried food, exercise more)	1.7 (± 0.7)	9.5
5	to find information on how to handle mental health problems? (e.g., stress, depression, anxiety)	2.1 (± 0.8)	26.9
6	to understand information about recommended health screenings or examinations (e.g., colorectal cancer screening, blood sugar test)?	1.8 (± 0.7)	12.6
7	to judge if information on unhealthy habits, such as smoking, low physical activity or drinking too much alcohol, are reliable (e.g., smoking causes cancer)?	1.7 (± 0.7)	9.8
8	to decide how you can protect yourself from illness using information from the mass media? (e.g., Newspapers, TV, Internet)	1.9 (± 0.7)	16.1
9	to find information on healthy lifestyles such as physical exercise, healthy food or nutrition?	1.8 (± 0.7)	9.8
10	to understand advice concerning your health from family or friends (e.g., healthy diet, regular exercise)?	1.8 (± 0.6)	8.4
11	to judge how your housing conditions may affect your health and well-being (e.g., light, ventilation)?	1.8 (± 0.7)	12.7
12	to make decisions to improve your health and well-being?	1.8 (± 0.7)	10.6
Total	HLS - Q12 score (continuous)	81.6 (23.0)	-
	HLS - Q12 score (categorical)^*^		
	Excellent	2940 (20.7)	-
	Sufficient	5216 (36.8)	-
	Problematic	4958 (35.0)	-
	Inadequate	1070 (7.5)	-

HLS, health literacy survey; SD, standard deviation. * (a) Excellent: “very easy” ≥ 50 and “very difficult” + “difficult” < 8.334, the number of answers with “very easy” should be above ½ and the answers for “very difficult” + “difficult” should be no more than 1/12; (b) Sufficient: “very easy” + “easy” > 83.33, at least 10 out of the 12 items should be answered with “very easy” or “easy” and not more than 2 out of 12 with “very difficult” or “difficult”; (c) Problematic: all respondents who were not in groups of “excellent”, “sufficient”, or “inadequate”; and (d) Inadequate: “very easy” < 8.334, “very difficult” and “difficult” ≥ 50, the number of answers with “very difficult” + “difficult” should be above ½ and for “very easy” should be no more than 1/12

### Reliability of the Chinese version of the HLS_19_-Q12 instrument

A Cronbach’s α of 0.93 showed excellent internal consistency for the Chinese version of the HLS_19_-Q12 instrument. The item-total correlation ranged from 0.67 to 0.81. Test-retest reliability was evaluated in 1298 respondents to whom the HLS_19_-Q12 instrument was administered twice, two weeks apart. The ICC was 0.54 (95% confidence interval (CI) = 0.49 to 0.58), indicating moderate test-retest reliability.

### Validity of the Chinese version of the HLS_19_-Q12 instrument

#### Content validity

By evaluating the relevance and comprehensiveness of each item for the construct of interest based on the theory-based matrix of the comprehensive multifaceted model of general health literacy [2], the translation committee ensured the content validity of the Chinese version of the HLS_19_-Q12.

### Structural validity

In terms of structural validity, we found that a single-factor model demonstrated good data fit: CFI = 0.94, TLI = 0.93, RMSEA = 0.095 (95% CI = 0.093, 0.097), suggesting strong structural validity. Factor loadings of each item are shown in Table 3.

**Table 3:** Factor loadings for the model reflecting the single latent construct of health literacy (*N* = 14,184)

Item	Factor loadings
1… to find out where to get professional help when you are ill? (e.g., doctor, pharmacist, psychologist)	0.68
2… to understand information about what to do in a medical emergency (e.g., fainting, coma, trauma)?	0.60
3… to judge the advantages and disadvantages of different treatment options (e.g., surgery, Western medicine, Chinese medicine)?	0.58
4… to act on advice from your doctor or pharmacist? (e.g., eat less fried food, exercise more)	0.71
5… to find information on how to handle mental health problems? (e.g., stress, depression, anxiety)	0.69
6… to understand information about recommended health screenings or examinations (e.g., colorectal cancer screening, blood sugar test)?	0.75
7… to judge if information on unhealthy habits, such as smoking, low physical activity or drinking too much alcohol, are reliable (e.g., smoking causes cancer)?	0.77
8… to decide how you can protect yourself from illness using information from the mass media? (e.g., Newspapers, TV, Internet)	0.81
9… to find information on healthy lifestyles such as physical exercise, healthy food or nutrition?	0.86
10… to understand advice concerning your health from family or friends (e.g., healthy diet, regular exercise)?	0.86
11… to judge how your housing conditions may affect your health and well-being (e.g., light, ventilation)?	0.81
12… to make decisions to improve your health and well-being?	0.83

### Concurrent predictive validity

Regarding the association between health literacy and social determinants of health (Table 4), we found that household income was the strongest predictor of health literacy, followed by highest educational level. When examining the association between health literacy and health consequences, we found that health literacy was the strongest predictor of health status, health-compromising behaviours, and health service use, compared to other social determinants of health such as household income. Participants with higher health literacy levels were likely to have better health status, fewer health-compromising behaviours, and less health service use.

**Table 4:** Results of concurrent predictive validity– Associations of health literacy with five social determinants of health and three health consequences (*N* = 14,184)

Predictor	Health literacy		Health status		Health compromising behaviours		Health service use	
	Unstandardised β (95% CI)	Standardised Beta	Unstandardised β (95% CI)	Standardised Beta	Unstandardised β (95% CI)	Standardised Beta	Unstandardised β (95% CI)	Standardised Beta
Health literacy (continuous)	-	-	-0.007 (-0.008, -0.007)	-0.21	-0.008 (-0.009, -0.007)	-0.13	-0.009 (-0.011, -0.008)	-0.12
Sex	0.47 (-0.55, 1.49)	0.01	-0.09 (-0.13, -0.06)	-0.05	1.17 (1.11, 1.22)	0.38	-0.16 (-0.24, -0.08)	-0.04
Age	-0.07 (-0.15, 0.02)	-0.02	0.01 (0, 0.01)	0.04	-0.03 (-0.04, -0.03)	-0.13	0.01 (0, 0.01)	0.02
Ethnicity	1.42 (-1.46, 4.31)	0.01	0.13 (0.03, 0.23)	0.03	-0.02 (-0.18, 0.14)	-0.002	0.43 (0.21, 0.64)	0.04
Highest educational level	0.94 (0.49, 1.39)	0.04	0.07 (0.06, 0.09)	0.10	0.06 (0.04, 0.09)	0.05	0.07 (0.04, 0.1)	0.04
Household income	1.82 (1.52, 2.13)	0.13	-0.01 (-0.02, 0)	-0.03	0.03 (0.02, 0.05)	0.04	-0.03 (-0.05, -0.01)	-0.03
Constant	75.36 (70.55, 80.17)	-	2.19 (2.02, 2.37)	-	5.18 (4.9, 5.45)	-	1.87 (1.5, 2.25)	-
Model fit (R^2^)	0.02		0.05		0.17		0.02	

Sex: Gender is male (versus female as a reference group). Age is continuous in years. Ethnicity is ethnic minorities (versus Han as a reference group). Highest educational level has five categories, including Year 6 or below, Year 7–9, Year 10–12, Diploma, and Bachelor’s degree or above. Household income has six categories, including 20,000 CNY or below, 20,000 to 39,000 CNY, 40,000 to 59,000 CNY, 60,000 to 79,000 CNY, 80,000 to 149,000 CNY, and 150,000 CNY or above. CI, confidence interval

### Convergent validity

The assessment of convergent validity showed weak to strong correlations between the HLS_19_-Q12 and each scale of the HLQ, with correlation coefficients ranging from 0.24 to 0.42 (see Appendix 5).

## Discussion

### Key summary of findings in the present study

This study validated the skills-based, comprehensive, and self-report HLS_19_-Q12 instrument in a large-scale general population of Chinese adults. Our findings showed that: (1) the HLS_19_-Q12 had excellent internal consistency and moderate test-retest reliability; (2) the HLS_19_-Q12 also showed satisfactory validity in terms of content validity, structural validity, concurrent predictive validity, and convergent validity.

Consistent with findings from the HLS_19_-Q12 validation study in the 17 European countries [22], we found similar patterns of item difficulties across the 12 items, ranging from 8.4 to 40.3% (compared to 8.1–43.0% in European countries), indicating that the tasks measured in the HLS_19_-Q12 are comparable between the Chinese context and European countries. For example, item 3 (“to judge the advantages and disadvantages of different treatment options”) was the most challenging one to respond in both contexts, with the highest percentage of participants answering “very difficult” or “difficult.” This corresponds to the highest percentage of participants who had missing data on item 3 in our sample. When comparing the total score of the HLS_19_-Q12, we had slightly higher average scores (81.6 ± 23.0) than those (76.0 ± 22.9) in the 17 European countries [22]. The total percentage of respondents who had sufficient or excellent levels of health literacy was similar between two contexts (China: 57.5%; All Europe countries: 55.0%).

The Cronbach’s α coefficient in our sample (0.93) was higher than that in the 17 European countries (ranging from 0.64 to 0.86) [22]. One possible reason might be that we added more contexts and examples (e.g., medical emergencies such as fainting, coma, trauma) in the content of several items, resulting in a clearer understanding to respondents. We also extend the current evidence by examining the test-retest reliability, which was not assessed in the European context. Our findings suggest that the HLS_19_-Q12 could be useful for repeated administration in future practice.

The HLS_19_-Q12 was developed based on the original HLS-EU-Q12 [22], which tested the 12-matrix (three health domains×four health management tasks) of general health literacy. Our CFA results showed that a single factor fitted the data well, supporting the HLS_19_-Q12 as a unidimensional instrument that measures general health literacy at the population level. Regarding concurrent predictive validity for the regression model with general health literacy as an outcome variable, we found that the R^2^ value (0.02) in our model was slightly lower than the average R^2^ value (0.07) in the 17 European countries [22]. Possible reasons for these discrepancies include different predictors involved in the regression model, sampling procedures, and administration modes (e.g., self-administered, face-to-face interviews). However, consistent with the HLS_19_-Q12 validation study in European countries [22] and in the USA [42], we found socioeconomic status such as education and income were the most important factor influencing general health literacy. For the regression models with general health literacy as a predictor variable and health status as an outcome, the R^2^ value (0.05) of our model was lower than that average R^2^ value (0.21) in the 17 European countries [22]. When comparing the standardised coefficient of each predictor, we found that general health literacy was the most important factor in our model and age was the most important factor in the model among 17 European countries [22], suggesting differences in the sample selection and other contextual factors may influence the results, as we targeted only school students’ primary carers and recruited them from only one city. Compared to the HLS_19_-Q12 validation study in European countries [22], we added evidence on the association between general health literacy and health-compromising behaviours and health service use. Our findings aligned with previous research showing that low health literacy was associated with poor health behaviours [43] and more health service use [44], indicating that health literacy is a strong social determinant of health.

Consistent with the a priori expectation, the HLS_19_-Q12 was correlated with the nine scales of HLQ, suggesting they may measure some common aspects of health literacy. However, there were differences in the effect size between each scale of HLQ. For example, Scale 1 (Feeling understood and supported by healthcare providers) had the lowest correlation (*r* = 0.24) and Scale 7 (Navigating the healthcare system) and 8 (Ability to find good health information) had the highest correlation (*r* = 0.42). The underlying item content between the HLS_19_-Q12 and HLQ may explain these differences, as the HLS_19_-Q12 did not ask any health-related task regarding social support whereas Scale 1 of the HLQ focused on the role of personal social support in health care.

### Strengths and limitations

While we used the STROBE and COSMIN checklist to enhance the reporting quality of this study, there are several limitations. First, we used convenience sampling to recruit primary carers of students who lived in urban or rural areas of Zhengzhou, Henan Province. In our sample, more than 70% of respondents were from rural areas, which may limit the generalisability of our findings. Participants were likely to be missed out if they were female, older, less educated, had lower income and lived in rural areas. Further validation of the HLS_19_-Q12 is needed with a more nationally representative sample of the Chinese population. In addition, further investigation such as using qualitative methods is needed regarding the patterns and reasons of missing data on each item of the HLS_19_-Q12 to ensure its suitability in the Chinese context. Second, measurement errors may exist for self-report instruments. Previous studies showed that respondents might overestimate their health literacy skills when using self-report items [45]. Future research is needed to investigate and compare the performance of the HLS_19_-Q12 and objective instruments (e.g., the task-based Newest Vital Sign). Third, the HLS_19_-Q12’s responsiveness and longitudinal predictive validity was not examined, which should be assessed in future longitudinal studies.

### Implications for future research and practice

Compared to the existing health literacy instruments (e.g., CHLS, HLQ) available in China, the Chinese version of the HLS_19_-Q12 has high potential to be easily administered in population surveys and program evaluations due to its strong psychometric properties and low administrative burden. The HLS_19_-Q12 could be a suitable instrument to measure and monitor health literacy over time at the population level, thus helping identify possible intervention opportunities to improve population health and reduce health inequities. In addition, the HLS_19_-Q12 might be useful for comparison of population health literacy levels between China and Western countries, thus contributing to a better understanding of Chinese adults’ health literacy in the global context.

We found that more than two fifths (42.5%) of our sample had problematic or inadequate health literacy when using the HLS_19_-Q12, suggesting an urgent need to promote and intervene on population health literacy. Recently, the Chinese government has issued the “Chinese Resident Ecological Environment and Health Literacy” and recommended using a whole-of-society approach to addressing limited health literacy and its impact on health outcomes among the general population. Given this study focused on the HLS_19_-Q12 validation, future research and practice are needed to explore possible pathways and intervention levers from a more precision public health perspective.

## Conclusion

The present study demonstrates that the Chinese version of the HLS_19_-Q12 is a reliable, valid, comprehensive instrument to measure health literacy in a large-scale population of adults from Zhengzhou in China. Further validation is needed to ensure the suitability of the HLS_19_-Q12 with a more nationally representative sample of Chinese adults. Compared to the existing knowledge-based or practice-based health literacy instruments used in China, the HLS_19_-Q12 is a skills-based and easy-to-administer health literacy instrument that could be easily integrated in population surveys and intervention evaluations to examine and monitor population health literacy levels. Our findings also suggest health literacy is both a social determinant of health and an intermediate health outcome. Promoting health literacy has the potential to improve population health and reduce health inequities.

### Electronic supplementary material

Below is the link to the electronic supplementary material.

Supplementary Material 1

## Data Availability

The datasets used during the current study are available from the corresponding author on reasonable request.

## Abbreviations

CFA: Confirmative Factor Analysis
CHLS: Chinese Health Literacy Scale
CI: Confidence Interval
COSMIN: COnsensus-based Standards for the selection of health status Measurement INstruments
HLQ: Health Literacy Questionnaire
HLS-EU-Q6: The 6-item European Health Literacy Survey Questionnaire
HLS-EU-Q12: The 12-item European Health Literacy Survey Questionnaire
HLS-EU-Q16: The 16-item European Health Literacy Survey Questionnaire
HLS-EU-Q47: The 47-item European Health Literacy Survey Questionnaire
HLS19 -Q12: The 12-item Health Literacy Population Survey
ICC: Intra-class Correlation Coefficient
REALM: Rapid Estimate of Adult Literacy in Medicine
RMSEA: Root Mean Square Error of Approximation
SAHL: The Short Assessment of Health Literacy
SD: Standard Deviation
STROBE: Strengthening the Reporting of Observational Studies in Epidemiology
TLI: Tucker and Lewis’s Index
WHO: World Health Organisation
