# Supplementary material for: Measuring General Health Literacy in Chinese adults: validation of the HLS19-Q12 instrument
Source: BMC Public Health. 2024 Apr 15;24:1036. doi: 10.1186/s12889-024-17977-1 (PMC11017570; doi:10.1186/s12889-024-17977-1)
Supplement: Supplementary file 1 — Supplementary Material 1 [file 12889_2024_17977_MOESM1_ESM.pdf]

Appendix 1. Minor changes to the HLS_{19} -Q12 when comparing English and Chinese versions

Item number	HLS_{19} -Q12		Notes
	English version	Chinese version	
1	…to find out where to get professional help when you are ill? (e.g., doctor, nurse, pharmacist, psychologist)	…to find out where to get professional help when you are ill? (e.g., doctor, pharmacist, psychologist, online medical consultation)	We removed the example of "nurse" and added an example of "online medical consultation."
2	…to understand information about what to do in a medical emergency?	…to understand information about what to do in a medical emergency (e.g., fainting, coma, trauma)?	We added three examples of medical emergencies (e.g., fainting, coma, trauma).
3	…to judge the advantages and disadvantages of different treatment options?	…to judge the advantages and disadvantages of different treatment options (e.g., surgery, Western medicine, Chinese medicine)?	We added three examples of different treatment options (e.g., surgery, Western medicine, Chinese medicine).
4	...to act on advice from your doctor or pharmacist?	...to act on advice (e.g., less fried food, more exercise) from your doctor or pharmacist?	We added two examples of advice from doctors or pharmacists (e.g., less fried food, more exercise).
5	…to find information on how to handle mental health problems? (e.g., stress, depression or anxiety)	…to find information on how to handle mental health problems? (e.g., stress, depression or anxiety)	No change
6	…to understand information about recommended health screenings or examinations? (e.g., colorectal cancer screening, blood sugar test)	…to understand information about recommended health screenings or examinations? (e.g., colorectal cancer screening, blood sugar test, blood pressure test)	We added an example of "blood pressure test".
7	...to judge if information on unhealthy habits, such as smoking, low physical activity or drinking too much alcohol, are reliable?	...to judge if information on unhealthy habits, such as smoking, low physical activity or drinking too much alcohol, are reliable? (e.g., smoking leads to cancer)	We added an example of "smoking leads to cancer".

Item number	HLS$_{19}$ -Q12		Notes
	English version	Chinese version	
8	…to decide how you can protect yourself from illness using information from the mass media? (e.g., Newspapers, TV or Internet)	…to decide how you can protect yourself from illness using information from the mass media? (e.g., Newspapers, TV or Internet)	No change
9	…to find information on healthy life styles such as physical exercise, healthy food or nutrition?	…to find information on healthy life styles such as physical exercise, healthy food or nutrition?	No change
10	…to understand advice concerning your health from family or friends?	…to understand advice concerning your health from family or friends (e.g., healthy food, more exercise)?	We added two examples of advice from family or friends (e.g., healthy food, more exercise).
11	...to judge how your housing conditions may affect your health and well-being?	...to judge how your housing conditions (e.g., light, ventilation, formaldehyde and other harmful gases) may affect your health and well-being?	We added three examples of housing conditions (e.g., light, ventilation, formaldehyde and other harmful gases).
12	…to make decisions to improve your health and well-being?	…to make decisions to improve your health and well-being?	No change

Appendix 2. Distribution of missing data in the analytic sample (N=14184)

Variable	Missing n (%)
Sex	0
Age	1340 (9.4)
Ethnicity	0
Single parent	0
Highest educational level	0
Household income	3542 (25.0)
Health Literacy Survey- Item 1	98 (0.7)
Health Literacy Survey- Item 2	557 (3.9)
Health Literacy Survey- Item 3	1256 (8.9)
Health Literacy Survey- Item 4	36 (0.3)
Health Literacy Survey- Item 5	644 (4.5)
Health Literacy Survey- Item 6	544 (3.8)
Health Literacy Survey- Item 7	90 (0.6)
Health Literacy Survey- Item 8	126 (0.9)
Health Literacy Survey- Item 9	30 (0.2)
Health Literacy Survey- Item 10	33 (0.2)
Health Literacy Survey- Item 11	139 (1.0)
Health Literacy Survey- Item 12	40 (0.3)
Health Literacy Survey- Total scores	0
General health status	0
Total scores of health-compromising behaviours	0
Total scores of health service use	0

Appendix 3. Results using multiply imputed data (N=14184)

Predictor	Health literacy		Global health status		Health compromising behaviours		Health service use	
	Unstandardised β (95% CI)	Standardised Beta	Unstandardised β (95% CI)	Standardised Beta	Unstandardised β (95% CI)	Standardised Beta	Unstandardised β (95% CI)	Standardised Beta
Health literacy (continuous)	-	-	-0.008 (-0.008, -0.007)	-0.21	-0.008 (-0.009, -0.007)	-0.13	-0.010 (-0.012, -0.009)	-0.14
Sex	0.74 (-0.14, 1.63)	0.01	-0.105 (-0.137, -0.074)	-0.05	1.134 (1.085, 1.184)	0.37	-0.132 (-0.198, -0.066)	-0.03
Age	-0.08 (-0.15, -0.01)	-0.02	0.006 (0.003, 0.008)	0.04	-0.032 (-0.036, -0.028)	-0.13	0.005 (-0.001, 0.01)	0.02
Ethnicity	1.84 (-0.64, 4.32)	0.01	0.146 (0.058, 0.234)	0.03	0.029 (-0.108, 0.167)	0.003	0.298 (0.114, 0.482)	0.03
Highest educational level	1.39 (1.02, 1.76)	0.07	0.070 (0.057, 0.084)	0.10	0.078 (0.057, 0.099)	0.06	0.041 (0.013, 0.068)	0.03
Household income	1.47 (1.19, 1.75)	0.10	-0.008 (-0.018, 0.002)	-0.02	0.024 (0.009, 0.04)	0.03	0.006 (-0.015, 0.026)	0.01
Constant	74.98 (70.88, 79.08)	-	2.196 (2.043, 2.348)	-	5.153 (4.915, 5.392)	-	2.103 (1.785, 2.422)	-
Model fit (R^2)	0.02		0.05		0.16		0.02	

Sex: Gender is male (versus female as a reference group). Age is continuous in years. Ethnicity is ethnic minorities (versus Han as a reference group). Highest educational level has five categories, including Year 6 or below, Year 7-9, Year 10-12, Diploma, and Bachelor's degree or above. Household income has six categories, including 20,000 CNY or below, 20,000 to 39,000 CNY, 40,000 to 59,000 CNY, 60,000 to 79,000 CNY, 80,000 to 149,000 CNY, and 150,000 CNY or above. CI, confidence interval.

Appendix 4. Missing data on health literacy

eTable 4.1. Summary of participants' characteristics by missing group

**Participants' characteristics**	**Missing data on health literacy**		
	**No (n=14184)**	**Yes (n=2003)**	**P value**
Location			
Urban	4013 (28.3)	355 (17.7)	P<0.001
Rural	10171 (71.7)	1648 (82.3)	
Sex			
Female	9863 (69.5)	1452 (72.5)	0.007
Male	4321 (30.5)	551 (27.5)	
Age group			
35 years or below	4647 (36.2)	631 (33.9)	P=0.001
36-40 years	4661 (36.3)	644 (34.5)	
41 years or above	3536 (27.5)	589 (31.6)	
Ethnicity			
Han	13816 (97.4)	1936 (96.7)	0.052
Ethnic minorities	368 (2.6)	67 (3.3)	
Single parent			
No	13487 (95.1)	1892 (94.5)	0.227
Yes	697 (4.9)	111 (5.5)	
Highest educational level			
Year 6 or below	556 (3.9)	214 (10.7)	P<0.001
Year 7-9	5138 (36.2)	1153 (57.6)	
Year 10-12	3581 (25.2)	390 (19.5)	
Diploma	2893 (20.4)	181 (9.0)	
Bachelor’s degree or above	2016 (14.2)	65 (3.2)	
Household income			
20,000 CNY or below	2833 (26.6)	694 (48.5)	P<0.001
20,000 to 39,000 CNY	2088 (19.6)	299 (20.9)	
40,000 to 59,000 CNY	1867 (17.5)	198 (13.8)	
60,000 to 79,000 CNY	1356 (12.7)	106 (7.4)	
80,000 to 149,000 CNY	1590 (14.9)	97 (6.8)	
150,000 CNY or above	908 (8.5)	37 (2.6)	

eTable 4.2. Distribution of missing data on each item among those who had missing data on the HLS19-Q12 (n=2003)

	n (%)
Item 1	
Non-missing	1459 (72.8)
Missing	544 (27.2)
Item 2	
Non-missing	862 (43.0)
Missing	1141 (57.0)
Item 3	
Non-missing	471 (23.5)
Missing	1532 (76.5)
Item 4	
Non-missing	1625 (81.1)
Missing	378 (18.9)
Item 5	
Non-missing	615 (30.7)
Missing	1388 (69.3)
Item 6	
Non-missing	569 (28.4)
Missing	1434 (71.6)
Item 7	
Non-missing	1115 (55.7)
Missing	888 (44.3)
Item 8	
Non-missing	1012 (50.5)
Missing	991 (49.5)
Item 9	
Non-missing	1292 (64.5)
Missing	711 (35.5)
Item 10	
Non-missing	1307 (65.3)
Missing	696 (34.7)
Item 11	
Non-missing	931 (46.5)
Missing	1072 (53.5)
Item 12	
Non-missing	1355 (67.6)
Missing	648 (32.4)

Appendix 5. Correlations between the 12-item Health Literacy Survey (HLS_{19}-Q12) and nine scales of the Health Literacy Questionnaire (HLQ-44)

Health Literacy Questionnaire (HLQ-44)	Health Literacy Survey (HLS_{19}-Q12)
Scale 1	0.24*
Scale 2	0.29*
Scale 3	0.25*
Scale 4	0.26*
Scale 5	0.27*
Scale 6	0.41*
Scale 7	0.42*
Scale 8	0.42*
Scale 9	0.39*

* p values < 0.001
